# A mathematical model of venous neointimal hyperplasia formation

**DOI:** 10.1186/1742-4682-5-2

**Published:** 2008-01-23

**Authors:** Paula Budu-Grajdeanu, Richard C Schugart, Avner Friedman, Christopher Valentine, Anil K Agarwal, Brad H Rovin

**Affiliations:** 1 Mathematical Biosciences Institute, The Ohio State University, Columbus, OH, USA; 2 Division of Nephrology, Department of Internal Medicine at The Ohio State University College of Medicine, Columbus, OH, USA

## Abstract

**Background:**

In hemodialysis patients, the most common cause of vascular access failure is neointimal hyperplasia of vascular smooth muscle cells at the venous anastomosis of arteriovenous fistulas and grafts. The release of growth factors due to surgical injury, oxidative stress and turbulent flow has been suggested as a possible mechanism for neointimal hyperplasia.

**Results:**

In this work, we construct a mathematical model which analyzes the role that growth factors might play in the stenosis at the venous anastomosis. The model consists of a system of partial differential equations describing the influence of oxidative stress and turbulent flow on growth factors, the interaction among growth factors, smooth muscle cells, and extracellular matrix, and the subsequent effect on the stenosis at the venous anastomosis, which, in turn, affects the level of oxidative stress and degree of turbulent flow. Computer simulations suggest that our model can be used to predict access stenosis as a function of the initial concentration of the growth factors inside the intimal-luminal space.

**Conclusion:**

The proposed model describes the formation of venous neointimal hyperplasia, based on pathogenic mechanisms. The results suggest that interventions aimed at specific growth factors may be successful in prolonging the life of the vascular access, while reducing the costs of vascular access maintenance. The model may also provide indication of when invasive access surveillance to repair stenosis should be undertaken.

## Background

### Vascular access dysfunction in chronic hemodialysis patients

Healthy kidneys filter wastes from blood and regulate electrolyte, acid-base, and volume homeostasis. When the kidneys fail, one needs treatment to replace the work the kidneys normally perform. One available treatment is hemodialysis, which utilizes an artificial kidney. The patients' blood is pumped into the artificial kidney where metabolic waste products diffuse out of the blood, and the cleansed blood is then returned back to the body. In order to perform hemodialysis, the patient must have suitable vascular access to allow adequate flow of blood to the hemodialysis circuit.

The most common types of vascular access used for hemodialysis are the arteriovenous (AV) fistula and the expanded polytetrafluoroethylene (ePTFE) graft. A surgeon creates an AV fistula by directly connecting an artery to a vein, usually in the forearm. The increased blood flow causes the vein to hypertrophy so that it can be used for repeated needle insertions. A graft connects an artery to a vein by using a synthetic tube of ePTFE, usually in the shape of a loop. It does not require as much time to mature as a fistula, so it can be used soon after placement. The direct purpose of the graft is to provide a vessel that is close to the skin (unlike the arteries) and has a high enough pressure to provide a sustained flow rate over 300 ml/min without collapsing (unlike the veins).

Both types of vascular access can have complications that require further treatment or surgery [1,2]. The data analysis of the Dialysis Outcomes Quality Initiative panel [2,3] suggests a primary patency of 85% for AV fistulas at one year and 75% at two years, whereas the ePTFE graft patency can be as low as 50% after one year and 20% at two years. These data exclude fistulae that did not mature adequately to support hemodialysis.

Over the last thirty years, hemodialysis vascular access dysfunction has been a major cause of morbidity and hospitalization among hemodialysis patients worldwide [4]. In the US alone, it is responsible for the hospitalization of more than 20% of patients with end-stage renal disease, at an annual cost of 1 billion dollars [2]. Novel monitoring and intervention programs, such as balloon angioplasty and surgery to open or bypass the stenosed segment, have improved the patency of native fistulae as well as ePTFE grafts, but at a significant financial cost. The expense of creating and maintaining vascular access for patients on dialysis accounts for a significant portion of any health care system. The intervention rates for ePTFE grafts are currently running six times higher than for fistulae [5]. While infections account for 10–15% of the failure of the ePTFE grafts, the leading cause of access failure is from loss of patency due to venous stenosis. Venous stenosis is the result of neointimal hyperplasia and luminal narrowing or occlusion [6-8], either at the site of venous anastomosis or in the downstream (proximal) vein. We assume that both AV fistulae and ePTFE grafts have similar mechanisms of venous neointimal hyperplasia. However, these accesses are inherently different with different flow characteristics. The model described here is more likely to be applicable to ePTFE grafts, rather than AV fistulae, due to exuberant inflammation produced by synthetic ePTFE graft.

### Pathogenesis of venous neointimal hyperplasia (VNH)

The most important events initiating the pathogenesis of VNH are: (a) surgical injury at the time of creation of the vascular access, as the vein is often stretched and manipulated; (b) hemodynamic stress at the graft-vein or artery-vein anastomosis, as a result of a combination of high shear stress and turbulence [2,9,10]; (c) the presence of the ePTFE graft itself, as a foreign body, which can attract macrophages that release cytokines and growth factors [2,11]; and (d) vascular access injury from dialysis needles. Other possible causes for VNH formation are: (e) differences in diameters between arteries and veins and less defined intimal layer may cause harmful fluid ebbs and backflow [2]; and (f) genetic predisposition of veins to vasoconstriction and neointimal hyperplasia after injury to endothelial and smooth muscle cells [12,13]. Treatment of an initial stenosis is often accomplished by balloon angioplasty. However, this treatment may inflict endothelial and smooth muscle cell injury, predisposing the vein to exaggerated VNH and repeated stenosis [2].

All the above stenosis-initiating events result in the activation of the smooth muscle cells and fibroblasts of the vascular media and adventitia, with migration into the intima and proliferation. In addition, there is a significant adventitial angiogenesis and excessive intimal synthesis of collagen [7,11]. This excess extracellular matrix (ECM) creates a neointimal expansion that contributes to access stenosis [14]. Access stenosis predisposes to access thrombosis and subsequently to access failure [15]. Thus, the so-called neo-intima is composed of vascular smooth muscle cells that are derived from all three layers of the vein.

Various groups [11,15-17] have demonstrated the expression of a number of chemical mediators during the pathogenesis of VNH, some of which could be potential therapeutic targets [2]. It has been demonstrated that (i) transforming growth factor-beta (TGF-*β*) stimulates smooth muscle cell growth and matrix production, and inhibits the degradation of matrix proteins [15,18,19]; (ii) platelet-derived growth factor (PDGF) has strong mitogenic and chemotactic effects on smooth muscle cells [7,20]; and (iii) endothelin-1 (ET-1) is a potent mitogenic peptide, and causes constriction of smooth muscle cells [16,21]. Each of these growth factors has been implicated in the occurrence of neointimal hyperplasia [16]. Several mechanisms have been suggested for enhanced production of these growth factors in neointimal hyperplasia including, in particular, oxidative stress [16] and turbulent flow [7,22].

Oxidative stress is characterized by circulating tissue proteins by oxidative activity [16]. Several studies have shown that increased levels of oxidative stress induce the production of TGF-*β *[16,23,24]. Other studies have implied that increased oxidative stress levels contribute to the platelet-activated release of PDGF and the production of ET-1 by endothelial cells [16,25,26].

It has also been suggested that turbulent flow of blood stimulates the mechanoreceptors on smooth muscle cells and shear-stress receptors on endothelial cells [27,28]. Turbulent flow might also stimulate the production of TGF-*β *since it is thought to be produced locally by smooth muscle cells as well as by macrophages and lymphocytes within the lesion created by the intimal hyperplasia [29]. Blood flow rate and the corresponding wall-shear stress can influence platelet aggregation, which, in turn, effects the production of PDGF [7,22,27]. Also, ET-1 levels increase in response to increased blood flow in the AV fistula [16,30].

### Present work

**Figure 1:**
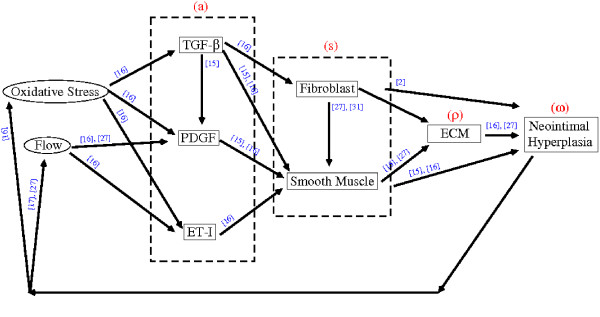
A schematic diagram illustrating some causes and effects of intimal hyperplasia. The red letters represent the variables in our model, while the blue numbers indicate the sources cited.

Based on the above cited work, a schematic diagram illustrating some causes and effects of VNH formation is represented in Figure 1. For simplicity, some of the intermediate factors are not included in the diagram. For example, we assume that fibroblasts produce basic fibroblast growth factors (bFGF) [31]; in turn, bFGFs stimulate the production of smooth muscle cells [27]. These two facts account for the arrow going from the fibroblast to smooth muscle cells (*i.e.*, the intermediate factor bFGF is dropped out). Also, the fibroblasts contribute to the intimal hyperplasia [2]. The fibroblasts in the neointima may acquire a smooth muscle cell-like phenotype by expressing smooth muscle actin, and thus be called myofibroblasts.

While the occurrence of VNH is well recognized, the pathogenesis of it is complex and still not well understood. Few studies have attempted to analyze the pathways that lead to dialysis access stenosis and direct attention to potential therapies [2,11]. Computational and mathematical tools have been applied to many areas of biology resulting in descriptive models with predictive capabilities. However, to our knowledge, there is no mathematical model to account for cellular and molecular interactions relevant to hemodialysis vascular access dysfunction. In the present work, we propose such a model for venous neointimal hyperplasia development describing:

• the interaction among growth factors, smooth muscle cells, and fibroblasts;

• the effect of these interactions on the venous stenosis;

• the effect of the stenosis on the level of oxidative stress and degree of turbulent flow;

• the influence of oxidative stress and turbulent flow on growth factors.

In the next section we introduce the mathematical model and illustrate how the model can potentially be used to predict vascular access failure based on the concentration of growth factors. The goal of any surveillance method is to detect access stenosis in a timely manner so that appropriate corrective steps can be undertaken prior to thrombosis. This is of critical importance, since the access survival after an episode of thrombosis is markedly reduced. With this in mind, we discuss possible applications of our results, not only to identify vascular access at the risk of thrombosis, but also for using the model to develop innovative strategies to prevent or delay vascular access failure. We conclude the work with comments on the mathematical model and future directions.

## Methods and Results

### Model description

The mathematical model that describes the VNH development is based on a simplification of the network diagram of Figure 1. However, we hope that the features retained for discussion are those of greatest importance in the present state of knowledge. The process of developing the model will identify important parameters and relationships that have not yet been investigated and can thus promote refinement in future studies.

To begin with, we identify the model variables and consider their movement, production and death in a radially symmetric control domain, Ω, that represents the intima and the lumen of the blood vessel at a cross-section where a stenosis develops. The geometry of the domain is specified by the radius *L *= *R*_0 _+ *d*_*INT*_, where *R*_0 _is the average radius of the lumen before the neointimal layer starts to form, and *d*_*INT *_stands for the average thickness of the venous intimal layer. In this setting, the boundary of the domain, Γ, corresponds to the interface between the media and the intima.

We now motivate our choice of the variables. For simplicity, we lump together several chemical species elemental to the process of neointima formation, as well as several cells and extracellular matrix components:

• *a*(*x*, *t*), general chemical species (TGF-*β*, PDGF, ET-1);

• *s*(*x*, *t*), general cellular species (smooth muscle cells, fibroblasts);

• *ρ*(*x*, *t*), extracellular matrix (collagen, fibronectin, elastin).

The quantity *a*(*x*, *t*) represents the concentration (in g/cm^3^) of growth factors at *x *∈ Ω in time *t*. In the absence of more detailed information on each factor, *a *accounts for all growth factors that potentially have a chemotactic effect on the cells. However, it is possible to separately describe the mechanism of action of particular growth factors as the model expands.

The quantity *s*(*x*, *t*) represents the density (in g/cm^3^) of cells at *x *∈ Ω in time *t*. We do not distinguish between various cells that are known to be involved in the formation of the neointimal hyperplasia, assuming instead that they all follow the same process of diffusion, chemotaxis and growth.

The quantity *ρ*(*x*, *t*) represents the density (in g/cm^3^) of extracellular matrix at *x *∈ Ω in time *t*. Although the matrix *ρ *and the cellular species *s *have different geometric features, for the purpose of this paper we assume that they both act as a source of material filling in the intimal-luminal space, and consequently we treat them in the same way.

To study the impact of the chemicals, cells and ECM on stenosis, we chose to monitor the reduction of the luminal volume *ω*(*t*), which is initially *ω*_0 _(according to clinicians, vascular access needs clinical intervention when the neointimal hyperplasia obstructs more than 50% of the initial luminal space, that is, when *ω*(*t*) = *ω*_0_/2). As the luminal space gets partially filled with cells *s *and extracellular matrix *ρ*, the boundary of the luminal space is not clearly defined. We take the point of view that the more material there is in the intimal-luminal domain, the smaller the luminal space will be, and simply define

$$\omega (t)=\omega_{0}-k\int_{\Omega }\left(s(x,t)+\rho (x,t)\right)dV.$$

where *k *is a dimensional constant.

Applying the laws of mass conservation to each of our variables we obtain the equations governing the evolution of *a*, *s *and *ρ*.

#### Chemical species

At the time *t *> 0 and the position *x *∈ Ω, the concentration of chemicals changes according to

$$\frac{\partial a}{\partial t}(x,t)=\underset{diffusion}{\underset{︸}{\nabla \left(D_{a}\nabla a\right)}}-\underset{removal}{\underset{︸}{\lambda \;a\;s}}+\underset{production}{\underset{︸}{c_{1}(\omega_{0}-\omega (t))}}.$$

We assume that the chemical species undergo random motion (*i.e.*, diffusion). Although the diffusion coefficient *D*_*a *_may in general depend on position, we take it here to be constant. Due to chemical signaling, the chemical species decrease through uptake by the cellular species. The value of the parameter *λ *is determined by the receptivity of cells to the growth factors. In the absence of more detailed information, we simply assume that the production rate of all growth factors is proportional to *ω*_0 _- *ω*(*t*). This term represents the observation that the production of chemical species depends on the oxidative stress and turbulent flow caused by the narrowing of the luminal space. We assume that the smaller the luminal space, the larger the oxidative pressure and shear flow, and also the larger the concentration of growth factors. Thus, the production of chemicals within the lesion is triggered by a large number of factors, which includes inflammation, hemodynamic and mechanical stresses.

#### Cellular species

The density of cells is assumed to follow the equation

$$\frac{\partial s}{\partial t}(x,t)=\underset{diffusion}{\underset{︸}{\nabla \left(D_{s}\nabla s\right)}}-\underset{chemotaxis}{\underset{︸}{\nabla \left(\chi_{a}\frac{\rho }{P}(1-\frac{s}{S})s\nabla a\right)}}+\underset{growth}{\underset{︸}{c_{2}s(1-\frac{s}{S})}}.$$

The cellular species undergo random motion, are chemotactically attracted to the chemicals in the presence of extracellular matrix, and grow up to a maximal value *S*. The chemotactic force is proportional to *s*∇*a*. We assume that the movement of cells due to chemotaxis cannot occur without extracellular matrix, which has maximum density *P*. For simplicity, the diffusion coefficient *D*_*s *_and the chemotactic coefficient *χ*_*a *_are considered constants. The parameter *c*_2 _of the logistic growth term depends on the whole family of growth factors, but for simplicity we have taken it to be constant. We note that in the expression for the chemotaxis we have lumped together all the cells (by *s*) and all the growth factors (by *a*). In an extended model one would quantify the effect of each specific growth factor on the proliferation of each cell type when the growth factors are separately modeled.

#### Extracellular matrix

We assume that extracellular matrix is being produced by cellular species, up to a maximum value *P*,

$$\frac{\partial \rho }{\partial t}(x,t)=\underset{growth}{\underset{︸}{c_{3}s(1-\frac{\rho }{P})}}.$$

We assume that the overproduction of extracellular matrix during the formation of VNH exceeds the degradation of the extracellular matrix, so that there is a total gain of the ECM density at rate *c*_3_, as long as the density is not saturated; for simplicity, we assume that *c*_3 _is constant.

#### Boundary and initial conditions

To complete the description of our model, it remains to specify the boundary and initial conditions for each of the variables. To begin with, we denote by *a*(*x*, 0) = *a*_0 _> 0 the initial concentration of growth factors in the proximal vein, at a cross-section characterized by the radius *R*(0) = *R*_0_. We further assume that no cellular species or extracellular matrix are present in the intimal-luminal space at time *t *= 0, hence *s*(*x*, 0) = 0 and *ρ*(*x*, 0) = 0.

If there is an influx of growth factors from the media-adventitia into the intima, we assume it is negligible compared to the production of the growth factors due to oxidative stresses and turbulent flow.

Consequently, we do not model the contribution of any factors from the medial-adventitial layers or nonvascular wall tissues, and therefore take

$${\nabla a\frac{x}{\left|x\right|}(x,t)|}_{x\in \Gamma }=0.$$

At low concentrations of chemicals inside the domain, there is no tendency for cells to cross the boundary into the intima. As the concentration of growth factors increase, a threshold concentration (*a *= *A*) is reached inside the domain, triggering the migration of cellular species from the medial-adventitial layers into the intima through the media-intima boundary. We assume a constant influx rate, *β*_*s*_, and write

$${\nabla s\frac{x}{\left|x\right|}(x,t)|}_{x\in \Gamma }=-\beta_{s}H(a-A),$$

although in a more general case, the rate of this influx of cells could depend on the concentration of chemicals. The term H(.) is the Heaviside step function, defined as *H*(*v*) = 0 when *v *< 0 and *H*(*v*) = 1 for *v *≥ 0, and it is used to represent the chemical signal that switches on as soon as the density arises above a threshold *A*.

Finally, to account for the inability of extracellular matrix to pass through the boundary, we impose a no-flux condition for *ρ*, namely

$${\nabla \rho \frac{x}{\left|x\right|}(x,t)|}_{x\in \Gamma }=0.$$

#### Parameter values

Table 1 gives a summary of the parameters and their numerical values used in the computer simulations to solve the PDE system (2)–(4) with the boundary conditions (5)–(7). The model parameters were obtained from a wide variety of experiments on many different human or animal models. Whenever such data were not available, we estimated the order of magnitude of the parameters and made choices that gave biologically reasonable results.

**Table 1:** Model parameters and their numerical values. Where no reference is given, the value chosen is our estimate.

	Description	Dimensional values	References
*D*_*a*_	Diffusion coefficient of chemicals	2.6 × 10^-7 ^cm^2 ^s^-1^	[32-34]
*D*_*s*_	Diffusion coefficient of cells	8 × 10^-10 ^cm^2 ^s^-1^	[35]
*C*_1_	Production rate of chemicals	0.01 cm^-4 ^s^-1 ^g	
*C*_2_	Proliferation rate of cells	0.0202 day^-1^	[36]
*C*_3_	Production rate of ECM by cells	0.01 h^-1^	
*λ*	Removal rate of chemicals	6.5 × 10^4 ^cm^3 ^s^-1 ^g^-1^	[37-39]
*χ*_*a*_	Chemotactic coefficient	2.9 × 10^2 ^cm^5 ^s^-1 ^g^-1^	[36],[40]
*A*	Intimal chemical concentration threshold	10^-9 ^g cm^-3^	[40]
*S*	Maximum cells density in O	1 g cm^-3^	
*P*	Maximum ECM density in O	1 g cm^-3^	
*R*_0_	Healthy vein luminal radius	1.35 mm	
*d*_*INT*_	Thickness of the venous intimal layer	0.15 mm	
*β*_*s*_	Cells movement across the boundary rate	0.2 cm^-4 ^g	
*k*	Neointima formation rate	0.02 g^-1 ^cm^3^	

### Model can be used to predict vascular access stenosis

In order to make predictions using the model described in the previous section, we numerically solve Eq. (2)-(4) for *a*_0 _= 25 × 10^-7 ^g cm^-3 ^and *R*_0 _= 1.35 mm. That is, when the lumen radius is 1.35 mm and the total concentration of growth factors inside the intimal-luminal cross-section of a vein is 25 × 10^-7 ^g cm^-3^. Given this initial data, we compute how the luminal radius changes over a year of dialysis treatment; for simplicity we do not take account of any effects caused by the actual treatment (*i.e.*, needle punctures).

In the 2-D case the lumen is a disc of radius *R*(*t*) and *ω*(*t*) = *πR*^2^(*t*). Since some important parameters are still currently unknown, initial understanding of the values of these parameters can be gained by doing the simulation in the simpler 1-D case. Furthermore, the results in the 1-D case already suggest strategies for delaying stenosis.

In the 1-D case, the lumen at each time *t *occupies an interval 0 <*x *<*R*(*t*), *ω*_0 _= *R*_0_, and *ω*(*t*) = *R*(*t*).

**Figure 2:**
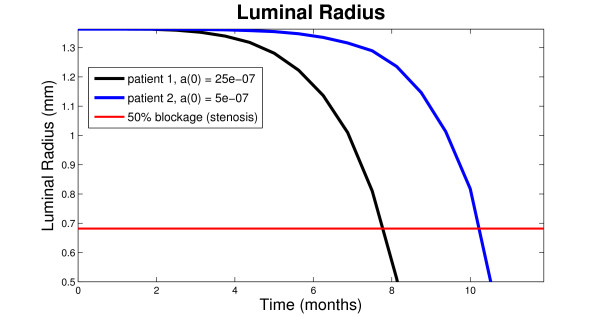
Numerical simulations showing that decrease of initial concentration of growth factors in the proximal vein, *a*(0), by a factor of 5 delays the onset of stenosis by more than 2 months.

The resulting time-dependent graph is the black curve shown in Figure 2. The red horizontal line marks the critical luminal radius associated with stenosis, here considered to be half of the luminal radius of a healthy vein. As time increases, the neointimal hyperplasia forms, decreasing the luminal radius. From this result, we see that the blockage caused by the formation of neointimal hyperplasia in a patient with 25 × 10^-7 ^g cm^-3 ^initial growth factors concentration will reach the critical stenosed state after approximately 8 months of dialysis.

### Impact of the growth factors on the vascular access lifespan

To better understand the effect of the concentration of growth factors on the development of VNH, we performed another numerical simulation, with a different input value for *a*_0_. We decrease the initial growth factors concentration from *a*_0 _= 25 × 10^-7 ^g cm^-3 ^to *a*_0 _= 5 × 10^-7 ^g cm^-3^. As before, we computed the changes in the luminal radius when the patient undergoes dialysis for over a year.

The result is the blue curve shown in Figure 2. We see that a drop in the initial concentration of growth factors delays the access stenosis by more than 2 months, prolonging the lifespan of the vascular access to more than 10 months. This implies that one mechanism by which the functional state of the hemodialysis vascular accesses can be extended is to control the concentration of the growth factors in the proximal vein. Our model and simulations, which build on cellular events leading to VNH formation, suggest that interventions aimed at specific chemical mediators involved in VNH formation may be successful in reducing the human and economic costs of vascular access dysfunction.

## Conclusion

The process of VNH formation is complex, involving a number of growth factors, different types of cells, ECM, oxidative stress, and fluid flow. Figure 1 illustrates the main interactions among these players. These interactions can be described in terms of a large system of partial differential equations. In this paper, we have developed a simple model in which we have lumped together all the chemical species into one variable, all the cellular species into one generic cell type, and treated the ECM as one concentration of connective tissue. We have also accounted for oxidative stress by having the growth factors increase as the luminal space decreases. Although our model is relatively simple, it captures some of the main features of VNH formation; in particular, it realistically predicts the stenotic event as a function of the initial concentration of the growth factors inside the intimal-luminal space.

### Future modeling opportunities

Our model represents a first step toward the development of a more realistic model that can be used by clinicians to identify vascular access at the risk of thrombosis, and to prevent or delay vascular access failure. Of the future modeling extensions needed to achieve this goal, perhaps the most important is a more accurate numerical approach. Rather than treating the stenotic lesion symmetrically in an 1-dimensional environment, in a future model we plan to develop higher dimensional numerical methods to investigate different geometries consistent with the complex nature of the VNH. Before embarking on a more detailed inspection of VNH formation it seems crucial to have a set of robust parameters. As only limited empirical data for various parameters is available at present, clinical studies need to be conducted in parallel with the development of the model to improve its reliability.

There are other different aspects of this project that can be improved, all aimed at better understanding of the cellular events leading to VNH formation. For simplicity, we have conglomerated all cytokines and cell types into one category, giving equal importance to all cytokines and cell types, which is unlikely to be true. However, with the current state of knowledge, it is not unreasonable to make such an assumption. As the relative importance of such factors is determined by future experiments, the model can be adjusted. Other issues that remain to be investigated concern: the contribution of chemicals from the medial and adventitial layers or from the nonvascular wall tissue; understanding the mechanical properties of the ECM building up the hyperplasia; understanding how the cells interact with the ECM; quantifying individual cell motion and cell-cell/cell-chemical interactions.

### Clinical relevance

With cooperative effort (*i.e.*, interplay between computational experiments and data) this model can be (expanded and) used by clinical researchers as a testbed for exploring and evaluating various therapies that can target both the traditional and the alternative pathways that are involved in the pathogenesis of VNH and vascular stenosis. In particular, our model suggests that clinical trials need to be conducted to examine the currently available agents that are known to inhibit the production of growth factors by smooth muscle cells, fibroblasts, or various other cells involved in the process of VNH formation.

Assuming this model is validated clinically, it could be applied in two main ways to address access function. First, because the model is predictive of 50% stenosis, it will provide an indication of when invasive access surveillance with the intention to repair stenosis should be undertaken. The model could be prospectively compared to current indicators of access intervention (declining flow rate, venous pressure) for predictive value, and the efficacy of repair on access lifespan could be determined for the model, and compared to current practices. This could be particularly useful in the case of patients that have undergone repeated access repair, since the current prognosis for such cases is less certain.

Second, because the model is based on pathogenic mechanisms, it can be used to design and test interventions that may prevent access stenosis. For example, methods to decrease oxidative stress could be prospectively tested to determine how they effect time to stenosis. Similarly, when clinically available, agents to reduce cytokine/growth factor expression in the access could be tested to determine how they extend the time to failure.

## Competing interests

The author(s) declare that they have no competing interests.

## Authors' contributions

PBG, RCS and AF formulated the model equations and wrote the manuscript. PBG performed the numerical calculations. CV, AKA and BHR were consulted on the model during the preparation of the paper, and all authors read and approved the manuscript.

## References

[B1] Asif A, Galadean F, Merrill D, Cherla G, Cipleu C, Epstein D, Roth D (2005). Inflow stenosis in arteriovenous fistulas and grafts: A multicenter, prospective study. Kidney Intl.

[B2] Roy-Chaudhury P, Sukhatme V, Cheung A (2006). Hemodialysis vascular access dysfunction: A cellular and molecular viewpoint. J Am Soc Nephrol.

[B3] Schwab S, Harrington J, Singh A, Roher R, Shohaib S, Perrone R, Meyer K, Beasley D (1999). Vascular access for hemodialysis. Kidney Int.

[B4] Feldman H, Kobrin S, Wasserstein A (1999). Hemodialysis vascular access morbidity. J Am Soc Nephrol.

[B5] NIH, NIDDK (2002). USDRS data report. Atlas of end-stage renal disease in the United States.

[B6] Sukhatme V (1996). Vascular access stenosis: Prospects for prevention and therapy. Kidney Int.

[B7] Swedberg S, Brown B, Sigley R, Wight T, Gordon D, Nicholls S (1989). Intimal fibromuscular hyperplasia at the venous anastomosis of PTFE grafts in hemodialysis patients: Clinical, immunocytochemical, light, and electron microscopic assessment. Circulation.

[B8] Windus D (1993). Permanent vascular access: A nephrologist's view. Am J Kidney Dis.

[B9] Remuzzi A, Ene-Iordache B, Mosconi L, Bruno S, Anghileri A, Antiga L, Remuzzi G (2003). Radial artery wall shear stress evaluation in patients with arteriovenous fistula for hemodialysis access. Biorheology.

[B10] Paszkowiak J, Dardik A (2003). Arterial wall shear stress: Observations from the bench to the bedside. Vasc Endovascular Surg.

[B11] Roy-Chaudhury P, Kelly B, Miller M, Reaves A, Armstrong J, Nanayakkara N, Heffelfinger S (2001). Venous neointimal hyperplasia in polytetrafluoroethylene dialysis grafts. Kidney Int.

[B12] Heine G, Ulrich C, Kohler H, Girndt M (2004). Is AV fistula patency associated with angiotensin-converting enzyme (ACE) polymorphism and ACE inhibitor intake?. Am J Nephrol.

[B13] Allon M, Robbin M (2002). Increasing arteriovenous fistulas in hemodialysis patients: Problems and solutions. Kidney Int.

[B14] Lemson M, Tordoir J, Daemen M, Kitslaar P (2000). Intimal hyperplasia in vascular grafts. Eur J Vasc Endovasc Surg.

[B15] Heine G, Ulrich C, Sester U, Sester M, Köhler K, Girndt M (2003). Transforming growth factor *β*1 genotype polymorphisms determine AV fistula patency in hemodialysis. Kidney Int.

[B16] Weiss M, Scivittaro V, Anderson J (2001). Oxidative stress and increased expression of growth factors in lesions of failed hemodialysis access. Am J Kidney Dis.

[B17] Mattana J, Effiong C, Kapasi A, Singhal P (1997). Leukocyte-polytetra-fluoroethylene interaction enhances proliferation of vascular smooth muscle cells via tumor necrosis factor-*α *secretion. Kidney Int.

[B18] Kaiura T, Itoh H, Kubaska S, McCaffrey T, Liu B, Kent K (2000). The effect of growth factors, cytokines, and extracellular matrix proteins on fibronectin production in human vascular smooth muscle cells. J Vasc Surg.

[B19] Border W, Noble N (1994). Transforming growth factor beta in tissue fibrosis. N Engl J Med.

[B20] Hehrlein C (1994). How do A-V fistula lose function? The roles of haemodynamics, vascular remodelling, and intimal hyperplasia. Nephrol Dial Transplant.

[B21] Masood I, Porter K, London N (1997). Endothelin-1 is a mediator of intimal hyperplasia in organ culture of human saphenous vein. Br J Surg.

[B22] Zamora J, Gao Z, Weilbaecher BG, Navarro L, Yves C, Hita C, Noon G (1985). Hemodynamic and morphologic features of ateriovenous angioaccess loop grafts. Trans Am Soc Artif Intern Organs.

[B23] Barcellos-Hoff M, Dix T (1996). Redox mediated activation of latent transforming growth factor-beta 1. Mol Endocrinol.

[B24] Leonarduzzi G, Scavazza A, Biasi F, Chiarpotto E, Camandola S, Vogl S, Dargel R, Poli G (1997). The lipid peroxidation end product 4-hydrox-2, 3-nonenol up-regulates transforming growth factor *β*1 expression in the macrophage lineage: A link between oxidative injury and fibrosclerosis. FASEB J.

[B25] Aviram M (1995). LDL-platelet interaction under oxidative stress induces macrophage foam cell formation. Thromb Haemost.

[B26] Miyauchi T, Masaki T (1999). Pathophysiology of endothelin in the cardiovascular system. Annu Rev Physiol.

[B27] Coulson A, Moya J (2000). Modification of venous end of dialysis grafts: an attempt to reduce neointimal hyperplasia. Dialysis Transplant.

[B28] Dzau V, Willerson J, Cohn J (1995). The role of vascular and structural remodeling. Cardiovascular Medicine.

[B29] Stracke S, Konner K, Kostlin I, Friedl R, Jehle P, Hombach V, Keller F, Waltenberger J (2002). Increased expression of TGF-beta1 and IGF-I in inflammatory stenotic lesions of hemodialysis fistulas. Kidney Int.

[B30] Wilkie M, Khandan-Nia N, Ghatei A, Bloom S, Raftery M, Cunningham J (1992). Does the arteriovenous fistula in chronic hemodialysis patients stimulate endothelin-1 release?. Nephrol Dial Transplant.

[B31] Akimoto S, Ishikawa O, Iijima C, Miyachi Y (1999). Expression of basic fibroblast growth factor and its receptor by fibroblast, macrophages and mast cells in hypertrophic scar. European Journal of Dermatology.

[B32] Brown D (1999). Dependence of neurones on astrocytes in a coculture system renders neurones sensitive to tranforming growth factor beta1-induced glutamate toxicity. J Neurochemistry.

[B33] Koka S, Vance J, Maze G (1995). Bone growth factors: Potential for use as an osseointegration enhancement techique (OIT). http://focus.hms.harvard.edu/2000/Sep15_2000/ophthalmology.html.

[B34] Woodcock E, Land S, Andrews R (1993). A low affinity, low molecular weight endothelin-A receptor present in neonatal rat heart. Clin Exp Pharmacol Physiol.

[B35] Chaplain M, Matzavinos A, Friedman A (2006). Mathematical modeling of spatio-temporal phenomena in tumor immunology. Tutorials in Mathematical Biosciences III: Cell Cycle, Proliferation, and Cancer.

[B36] Olsen L, Sherratt J, Maini P (1995). A mechanochemical model for adult wound contraction and the permanence of the contracted tissue displacement profile. J Theor Biol.

[B37] Stacker S, Stenvers K, Caesar C, Vitali A, Domagala T, Nice E, Roufail S, Simpson R, Moritz R, Karpanen T, Alitalo K, Achen M (1999). Biosynthesis of vascular endothelial growth factor-D involves proteolytic processing which generates non-covalent homodimers. J Biol Chem.

[B38] Mäkinen T, Veikkola T, Mustjoki S, Karpanen T, Catimel B, Nice E, Wise L, Mercer A, Kowalski H, Kerjaschki D, Stacker S, Achen M, Alitalo K (2001). Isolated lymphatic endothelial cells transduce growth, survival, and migratory signals via the VEGF-C/D receptor VEGFR-3. EMBO J.

[B39] Baldwin M, Catimel B, Nice E, Roufail S, Hall N, Stenvers K, Karkkainen M, Alitalo K, Stacker S, Achen M (2001). The specificity of receptor binding by vascular endothelial growth factor-D is different in mouse and man. J Biol Chem.

[B40] Grotendorst G, Cohen I, Diegelmann R, Lindblad W (1992). Chemoattractants and growth factors. Wound Healing: Biochemical and Clinical Aspects.

